# Cytotoxic and potent CYP1 inhibitors from the marine algae *Cymopolia barbata*

**DOI:** 10.1186/2191-2858-2-21

**Published:** 2012-06-11

**Authors:** Simone Badal, Winklet Gallimore, George Huang, Tzuen-Rong Jeremy Tzeng, Rupika Delgoda

**Affiliations:** 1 Natural Products Institute, Faculty of Pure and Applied Sciences, University of the West Indies, Mona, West Indies, Jamaica; 2 Department of Chemistry, Faculty of Pure and Applied Sciences, University of the West Indies, Mona, West Indies, Jamaica; 3 Department of Biological Sciences, Clemson University, Clemson, SC 29634, USA

**Keywords:** *Cymopolia barbata*, Dasycladaceae, Anticancer, CYP450, Chemoprevention, Chemoprotection.

## Abstract

**Background:**

Extracts from the marine algae *Cymopolia barbata* have previously shown promising pharmacological activity including antifungal, antitumor, antimicrobial, and antimutagenic properties. Even though extracts have demonstrated such bioactivity, isolated ingredients responsible for such bioactivity remain unspecified. In this study, we describe chemical characterization and evaluations of biological activity of prenylated bromohydroquinones (PBQ) isolated from the marine algae *C. barbata* for their cytotoxic and chemopreventive potential.

**Methods:**

The impact of PBQs on the viability of cell lines (MCF-7, HT29, HepG, and CCD18 Co) was evaluated using the MTS assay. In addition, their inhibitory impact on the activities of heterologously expressed cytochrome P450 (CYP) enzymes (CYP1A1, CYP1A2, CYP1B1, CYP2C19, CYP2D6, and CYP3A4) was evaluated using a fluorescent assay.

**Results:**

7-Hydroxycymopochromanone (PBQ1) and 7-hydroxycymopolone (PBQ2) were isolated using liquid and column chromatography, identified using ^1^ H and ^13^ C NMR spectra and compared with the spectra of previously isolated PBQs. PBQ2 selectively impacted the viability of HT29, colon cancer cells with similar potency to the known chemotherapeutic drug, fluorouracil (IC_50,_ 19.82 ± 0.46 μM compared to 23.50 ± 1.12 μM, respectively) with impact toward normal colon cells also being comparable (55.65 ± 3.28 compared to 55.51 ± 3.71 μM, respectively), while PBQ1 had no impact on these cells. Both PBQs had potent inhibition against the activities of CYP1A1 and CYP1B1, the latter which is known to be a universal marker for cancer and a target for drug discovery. Inhibitors of CYP1 enzymes by virtue of the prevention of activation of carcinogens such as benzo-a-pyrene have drawn attention as potential chemopreventors. PBQ2 potently inhibited the activity of CYP1B1 (IC_50_ 0.14 ± 0.04 μM), while both PBQ1 and PBQ2 potently inhibited the activity of CYP1A1 (IC_50_s of 0.39 ± 0.05 μM and 0.93 ± 0.26 μM, respectively). Further characterizations showed partial noncompetitive enzyme kinetics for PBQ2 with CYP1B1 with a *K*_*i*_ of 4.7 × 10^–3^ ± 5.1 × 10^–4^ μM and uncompetitive kinetics with CYP1A1 (*K*_*i*_ = 0.84 ± 0.07 μM); while PBQ1 displayed partial non competitive enzyme kinetics with CYP1A1 (*K*_*i*_ of 3.07 ± 0.69 μM), noncompetitive kinetics with CYP1A2 (*K*_*i*_ *=* 9.16 ± 4.68 μM) and uncompetitive kinetics with CYP1B1 (*K*_*i*_ = 0.26 ± 0.03 μM) .

**Conclusions:**

We report for the first time, two isolated ingredients from *C. barbata*, PBQ1 and PBQ2, that show potential as valuable chemotherapeutic compounds. A hydroxyl moiety resident in PBQ2 appears to be critical for selectivity and potency against the cancer colon cells, HT29, in comparison to the three other malignant cell lines studied. PBQs also show potency against the activities of CYP1 enzyme which may be a lead in chemoprevention. This study, the first on isolates from these marine algae, exemplifies the value of searching within nature for unique structural motifs that can display multiple biological activities.

## Background

**Figure 1:**
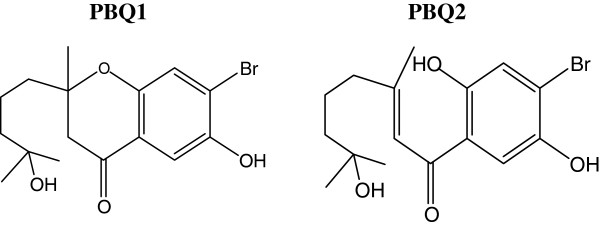
**General structures of polyisoprenylated bromohydroquinones (PBQ1 and PBQ2) isolated from the marine algae, *C. barbata.***

*Cymopolia barbata* (Linnaeus) V.Lamouroux (Dasycladaceae) is widespread in shallow waters and is seen covering rocks by the shorelines in tropical marine habitats. Known to grow to about 10-cm high, these green algae (Chlorophyta) have tufts at the end of their stems that are lightly calcified. Extracts from this plant have previously shown significant pharmacological properties such as antifungal, antitumor, antimicrobial, and antimutagenic activities [1-8]. Although the cymopols are known halogenated natural products which have been isolated from *C. barbata*, active ingredients responsible for the displayed biological activities remain unspecified. In this study, we investigated bioactivities of prenylated bromohydroquinones (PBQ), cymopol-related metabolites which are known to accumulate in *C. barbata*, and report, for the first time, biological activities from single ingredients isolated from this marine algae. Two of these compounds namely, 7-Hydroxycymopochromanone (PBQ1) and 7-hydroxycymopolone (PBQ2), shown in Figure 1 were investigated for cytotoxicity against three cancerous cell lines, one normal cell line, in addition to their potential for chemoprevention via inhibition of cytochrome P450 (CYP) 1 enzymes.

The CYP1 family of enzymes and in particular CYP1B1 appears to be a universal molecular cancer marker and a target for drug discovery. Findings of the over-expression of CYP1B1 in many tumor tissues compared with normal surrounding cells have led to the search for prodrugs reliant on CYP1B1 metabolism for the conversion into cytotoxic therapeutics [9]. The modification in the expression levels of CYP1B1 has been shown to modulate tumor progression [10] and thus specific inhibitors are expected to be of therapeutic/preventive benefit. Further, the involvement of CYP1 enzymes in the bioactivation of procarcinogens such as polycyclic aromatic hydrocarbons (PAHs), heterocyclic amines, aromatic amines, and nitro polycyclic hydrocarbons [11], in addition to the biotransformation of anticancer drugs, has stimulated research into inhibitors of CYP1 enzyme activity [12,13]. Such inhibitors are thought to be potential anti-carcinogens if they could inhibit the activities of CYP1B1 and CYP1A1 to metabolize PAHs to toxic intermediates and/or decrease their ability to detoxify cancer drugs. A number of natural products have been found to be direct inhibitors of CYP1 enzymes, as well as generate metabolites that are CYP inhibitors with cytotoxic properties. In the study described in this article, PBQ2 demonstrated potent inhibition against CYP1B1 activity, together with promising and specific activity against the colon cancer cell line HT29. The examination of a close structural relative, PBQ1, also allows identification of structural motifs critical for biological activity.

## Methods

### Chemicals

All chemicals for the MTS and CYP inhibition assays were purchased from Sigma-Aldrich (St. Louis, MO, USA). All CYP substrates and metabolites were purchased from Gentest Corporation (Worburn, MA, USA).

### Cell lines and CYP microsomes

All cell lines along with their respective media and supplements were purchased from ATCC (Manassas, VA, USA). *Escherichia coli* membranes expressing human CYP1A1, CYP1A2, CYP1B1, CYP2D6, CYP3A4, and CYP2C19 co-expressed with CYP reductase were purchased from Cypex Ltd. (Dundee, UK).

### Cell culture and cytotoxicity assays

Cell lines (CCD18 Co, HepG2 and MCF-7) were maintained in ATCC-formulated Eagle’s Minimum Essential Medium and HT29 was maintained in McCoy’s 5a Modified Medium supplemented with 10% fotal bovine serum (Atlas; Fort Collins, CO, USA), 10 mM HEPES solution, 100 mM l-glutamine penicillin streptomycin solution, 3 g/L glucose, and 1.5 g/L of sodium bicarbonate. Cells were maintained at 37°C with 5% CO_2_ in Corning 75 cm^2^ culture flasks. Cells were exposed to a given isolate or known anticancer agent for 24 h. Following the appropriate treatments, cell viability was evaluated using an MTS assay according to the manufacturer’s instructions [14]. All assays were performed at least three times and were monitored spectrophotometrically at 590 nm [15]. Cell viability was recorded as percentage relative to vehicle solvent-treated control.

### CYP inhibition assays

The test compounds were evaluated for their ability to inhibit the catalytic activity of human CYP1 enzymes by means of high throughput fluorometric detection assays conducted in 96-well microtitre plates as described elsewhere [16,17]. 7-Ethoxyresorufin (ERes) was used as a substrate for detecting activity of CYP1B1 and 7-ethoxy-3-cyanocoumarin (CEC) was used as a substrate for both CYP1A1 and CYP1A2. Further, the substrates, 3-[2-(*NN*-diethyl-*N*-methylamino)ethyl]-7-methoxy-4methylcoumarin (AMMC), 7-benzyloxy-4-trifluoromethylcoumarin (BFC), and CEC were used as substrates for CYP2D6, CYP3A4, and CYP2C19, respectively. The reactions were monitored fluorometrically at 37°C, using a Varian Cary Eclipse fluorescence spectrophotometer. All inhibitors were dissolved in a solvent of 20% acetonitrile in water and less than 0.3% of acetonitrile was used in the final assay.

### Data analysis

IC_50_ and *K*_*i*_ values were determined by fitting the data in Sigma Plot (version 10.0) and enzyme kinetics module, using nonlinear regression analysis. The apparent *K*_*i*_ values were determined on the basis of visual inspection of Eadie-Hofstee and various statistics to evaluate goodness of fit, such as the size of the sum of squares of residuals, Akaike information criterion, and standard error (Enzyme kinetics module, version 1.3). The data listed represent the average values from three different determinations.

## Results and discussion

**Figure 2:**
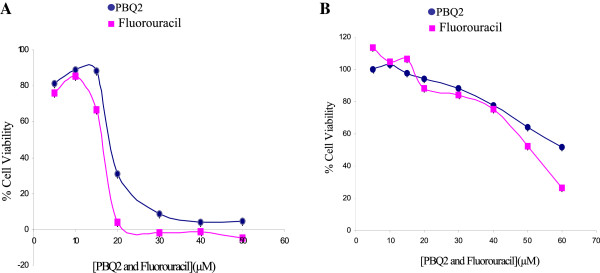
Percentage cell viability of colon cancer cells (HT29; A) and normal colon cells (CCD18 Co; B) in the presence of PBQ2 and known chemotherapeutic drug fluorouracil.

Two PBQs (Figure 1) were isolated from the marine alga *C. barbata* and investigated for biological activity. The ability of these compounds to interfere with the reduction of the tetrazolium salt in the MTS assay was examined as a measure of impact on cell viability (Figure 2) using normal and cancer colon cells (CCD18 Co and HT29, respectively) along with liver and breast cancer cells (HepG2 and MCF-7, respectively). IC_50_ values were calculated for test compounds and positive control known drug entities, doxorubicin, fluorouracil, and tamoxifen (Table 1). PBQ2 selectively impacted the viability of colon cells, HT29 with comparable potency to fluorouracil (for HT29 cancer cells: IC_50_, 19.82 ± 0.46 μM compared to 23.50 ± 1.12 μM and normal colon cells, CCD18 Co IC_50_, 55.65 ± 3.28 compared to 55.51 ± 3.71 μM, respectively). PBQ1 had no significant impact (<10% at 60 μM) on any of the cell lines tested.

**Table 1:** **IC**_**50**_**values (µM) obtained from the interaction of isomers of PBQs with colon cancer cell line (HT29) and the normal colon cell line (CCD18Co) along with positive controls**

**Compound**	**Cell lines**			
	**CCD18 Co**	**HT29**	**HepG**	**MCF-7**
PBQ1	NI	NI	NI	NI
PBQ2	55.65 ± 3.28	19.82 ± 0.46	NI	NI
Tamoxifen	NA	NA	NA	17.28 ± 0.06
Fluorouracil	55.51 ± 3.71	23.50 ± 1.12	ND	ND
Doxorubicin	NA	NA	18.61 ± 0.58	NA

*Key* NI, no impact (<10% inhibition at 60 μM); NA, not applicable; ND, not determined.

To verify the accuracy of experimental techniques employed to detect CYP inhibition, assays with known inhibitors were carried out with furafylline (against CYP1A2 activity), ketoconazole (against activities of CYP1A1, CYP1B1, and CYP3A4), (−)-*N*-3-Benzyl-phenobarbital (against 2 C19) and quinidine (against CYP2D6 activity) and the obtained IC_50_ values (0.8 ± 0.2, 0.04 ± 0.01, 6.3 ± 1.7, 0.06 ± 0.01, 0.3 ± 0.01, 0.03 ± 0.01 μM, respectively) compared well with published values (0.99, <10, <10, 0.06, 0.25, and 0.04 μM, respectively; [17-20]). Michaelis constant, *K*_M_, was determined for each marker substrate under the specified experimental conditions, in order to determine suitable substrate concentrations for assessing inhibitory potential of test compounds [21].

**Figure 3:**
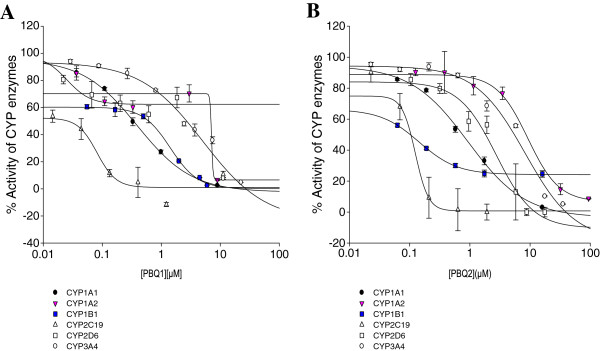
**Inhibition of activities of CYP isoforms by PBQs 1 (A) and PBQ2 (B).** Human recombinant CYP1B1-catalyzed dealkylation of ERes (0.37 μM), CYP1A1, CYP1A2, and CYP2C19-catalyzed dealkylation of CEC (0.5, 5, and 25 μM, respectively) CYP2D6-catalyzed dealkylation of AMMC (1.5 μM) and CYP3A4-catalyzed debenzylation of BFC (50 μM) were determined in the presence of varying concentrations of PBQs ranging between 0 and 900 μM, as described in the section “methods”. Control enzyme activity (mean ± SEM) for CYP1B1, CYP1A2, CYP1A1, CYP2C19, CYP2D6, and CYP3A4 was 0.34 ± 0.08, 0.23 ± 0.04, 0.86 ± 0.01, 0.25 ± 0.02, 0.10 ± 0.003, and 1.28 ± 0.07 μM/min/pmol of CYP, respectively. Data are expressed as mean percentage of control enzyme activity for three independent experiments.

**Figure 4:**
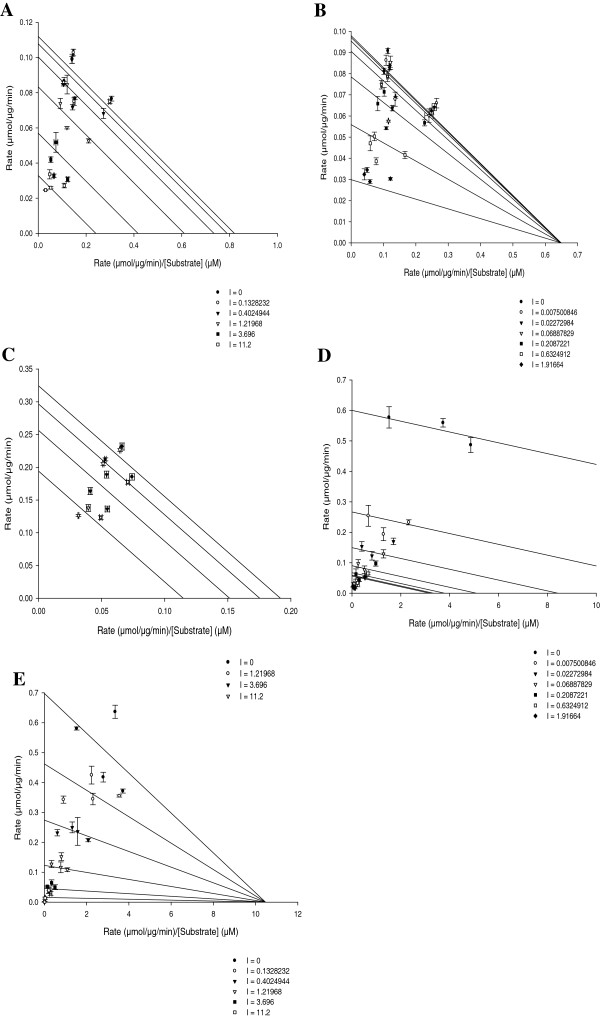
**Eadie-Hofstee plots for inhibition of activities of CYP1A1 (A), CYP1A2(B), and CYP1B1(C) by PBQ1 along with inhibition of activities of CYP1A1 (D) and CYP1B1 (E) by PBQ2.** CEC dealkylation catalyzed by recombinant CYP1A1 and CYP1A2 was determined in the absence and presence of six different concentrations of PBQs along with ERes dealkylation catalyzed by recombinant CYP1B1. Each point represents the mean ± SEM of three independent experiments.

Both PBQs 1 and 2 potently (IC_50_ < 1 μM) inhibited the activity of CYP1A1 (IC_50_s of 0.39 ± 0.05 and 0.93 ± 0.26 μM, respectively). PBQ2 also potently inhibited the activity of CYP1B1 (IC_50_, 0.14 ± 0.04 μM) as shown in Figure 3. For those interactions yielding an IC_50_ < 10 μM against the activities of CYP1 family, further kinetic characterization was carried out to determine the nature of the inhibition, and Eadie-Hofstee plots are illustrated in Figure 4. Reversible enzyme kinetics was observed for PBQ1 with partial noncompetitive inhibition of CYP1A1 activity, noncompetitive inhibition of CYP1A2 activity (*K*_*i*_s of 3.07 ± 0.69 and 9.16 ± 4.68 μM, respectively) and uncompetitive inhibition of CYP1B1 activity (*K*_*i*_ of 0.26 ± 0.03 μM). PBQ2 displayed uncompetitive inhibition of the activity of CYP1A1 (*K*_*i*_ of 0.84 ± 0.07 μM) and partial noncompetitive inhibition of the activity of CYP1B1 (*K*_*i*_ of 4.7 × 10^–3^ ± 5.1 × 10^–4^ μM).

Further characterization of the isolated test compounds against other major drug metabolizing P450 enzymes (CYP2C19, CYP2D6, and CYP3A4) was carried out. A summary table with all IC_50_ data is presented in Table 2. As seen therein, while both PBQs 1 and 2 moderately (IC_50_ > 1 μM) inhibited the activities of CYP2D6 (IC_50_s, 1.03 ± 0.40 and 2.75 ± 0.96 μM, respectively) and CYP3A4 (IC_50_s, of 5.07 ± 3.54 and 8.31 ± 4.67 μM, respectively). PBQ1 and PBQ2 potently inhibited the activity of CYP2C19 (0.08 ± 0.03 and 0.12 ± 0.06 μM, respectively).

**Table 2:** **IC**_**50**_**values (µM) obtained from the interaction of isomers of PBQs with CYP enzymes**

**Compounds**	**CYP I isoforms**					
	**1A1**	**1A2**	**1B1**	**2 C19**	**2D6**	**3A4**
PBQ1	0.39 ± 0.05	9.75 ± 0.0365	1.42 ± 0.14	0.08 ± 0.03	1.03 ± 0.40	5.07 ± 3.54
PBQ2	0.93 ± 0.26	10.55 ± 6.75	0.14 ± 0.04	0.12 ± 0.06	2.75 ± 0.96	8.31 ± 4.67

From the panel of cell lines tested, the impact on the viability of malignant colon (HT29) and normal colon (CCD18 Co) cells by PBQ2 was similar to that imparted by the chemotherapeutic drug, fluorouracil, with comparable IC_50_ values. PBQ2 also displayed selective cytotoxicity toward HT29 cells with no impact on cancerous liver (HepG2) and breast (MCF-7) cells. PBQ1, its structural isomer on the other hand, had no impact on any of the cell lines investigated and thus the presence of a tertiary hydroxyl group on PBQ2 appears to be critical for the observed bioactivity. Be it the formation of hydrogen bonds with key residues within the cell or during receptor-mediated cell permeability, the conjugated, open ring, and hydroxyl group presence in PBQ2 plays a crucial role in impacting cell viability compared with epoxy moiety of PBQ1.

Inhibitors of CYPs1A1 and 1B1 enzymes have received particular interest due to their role in reducing the activation of carcinogens and thus as chemoprotectors and chemotherapeutics. Several classes of natural compounds [22], including flavonoids [23,24] and organosulfur compounds [13,25,26], have demonstrated great potential in chemoprevention and thus provide the impetus for the search for others. Both PBQs potently (IC_50_ < 1 μM) inhibited the activity of CYP1A1while PBQ2 also displayed potency toward CYP1B1. Studies using knock-out mice models have linked CYP1B1 with the activation of several carcinogens, such as B*a*P and DMBA [11] and have also been shown to play an important role in modulating tumor progression [10,27]. Thus, the potency toward both CYP1B1 and CYP1A1 activities by PBQ2 suggests potential chemopreventive bioactivity in vivo. Conversely, PBQ1 with demonstrated CYP1A1 inhibition, devoid of cytotoxicity on all cells examined in this study, also highlights it as an attractive candidate with potential for development as a chemoprotector.

The development of novel classes of therapeutics that can target both drug metabolizing enzymes and disease pathways is a multi-targeted approach that may well suit the multi-factorial origins of a disease such as cancer. Examples of such compounds include isothiocyanate which impact both Nrf-2 transcription factors, and inhibit the nuclear factor ĸß pathway to exhibit potent anti-inflammatory properties [28]. Such attractive dual qualities are displayed by PBQ2 in this study, with potent and selective targeting of HT29 colon cancer cells, as well as the inhibition of CYP1A1 and CYP1B1enzyme activities.

The potency of PBQ2 can be put into perspective with the reported inhibitory effect of eight flavonoids tested against recombinant human CYP1B1 and CYP1A1 enzyme activities which showed a range in IC_50_s between 0.3 and 27 μM [29]. They made ideal chemoprotectants against prostate cancer [30]. PBQ2 had an IC_50_ of 0.14 μM, which appears to be more potent at inhibiting CYP1B1 activity than all the eight flavonoids tested by Chaudhary and Willet [29], making this isolate an ideal candidate for further research.

PBQs examined in this study displayed reversible, non or uncompetitive (partial or full) kinetics on CYP1 enzyme activities. In noncompetitive inhibition, typically the IC_50_ value is equal to the *K*_*i*_, while in uncompetitive inhibition IC_50_ will equal twice the value of the *K*_*i*_ for experiments where the substrate concentration is close to the *K*_m_ value [31], as designed in our experiments. Such approximations are observed in the kinetics of PBQ1 with CYPs1A2, 1B1 and of PBQ2 with CYP1A1, although deviations from these approximations were seen for interactions of PBQ1 with CYP1A1 and PBQ2 with CYP1B1 where the partial noncompetitive kinetics were observed and such mixed type binding may complicate relations between IC_50_ and *K*_*i*_s. Due to the non and uncompetitive nature of PBQ binding with CYP1A1, previous active site models developed for CYP1A1 using natural product quassinoids [20,32], were therefore not useful in shedding light on structure–activity relationships within the active site. The dietary flavonoid, galangin, shown to display inhibition of DMBA-induced CYP1A1 in MCF-7 breast cancer cells, was of a noncompetitive, dose-dependent manner [33] similar to that of PBQ1.

Investigating the impact of these PBQs against the other major drug metabolising enzymes (CYP2C19, CYP2D6, and CYP3A4) allowed for the predictions of drug interaction potential. The impacts on CYP3A4 and CYP2D6, the enzymes responsible for metabolism of over 90% of drugs on the market, were only moderate by the two PBQs, suggesting unlikely metabolism-based drug interactions via these important enzymes. However, both compounds potently inhibited CYP2C19 activity. CYP2C19 is also involved in the process of carcinogenesis *albeit* with lower impact than the CYP1 family, and the CYP2C19 inhibition by the PBQs may prove useful in chemopreventive value, although drug interactions possibility via this enzyme that metabolises important therapeutics such as omeprazole and theophylline will remain a concern compounded by likely variations in inhibition reliant on the expression levels of this polymorphic enzyme.

### Experimental

#### *Plant material*

*Cymopolia barbata* was collected from the shoreline of the north eastern coast of Jamaica at Fairy Hill Beach in the parish of Portland at a depth of 0.5 m in June 2004. A voucher specimen (#UWI-Mona 35, 438) was deposited in the Herbarium at the University of the West Indies, Mona, Jamaica.

#### *Extraction and isolation*

The air-dried sample (962.15 g) was extracted with methanol:dichloromethane (1:1) to yield a dark green gum (30.91 g), a portion of which (12.8 g) was subjected to vacuum liquid chromatography on silica gel in a 2-L sintered funnel with a gradient elution system consisting of increasing proportions of CH_2_Cl_2_ in hexanes, 100% CH_2_Cl_2_ with final elution in 20% methanol:CH_2_Cl_2_. Of the 56 fractions obtained, fraction 22–24 (2.35 g), which eluted in 20% methanol:CH_2_Cl_2_, underwent further gravity column chromatography to afford 172 sub fractions. From this column, sub fraction 20–22 was found to contain 7-hydroxycymopochromanone (PBQ1). Another portion of the crude extract (2.0130 g) was subjected to column chromatography on a Sephadex LH-20 column in methanol resulting in 11 main combined fractions. The fifth combined fraction from this column was subjected to silica gel chromatography in 30% acetone:hexane to afford 37 fractions. Fraction 23–28 was found to contain 7-hydroxycymopolone (PBQ2). Both PBQs were identified by comparison of their ^1^ H and ^13^ C NMR data with the literature [1,34].

##### *NMR data for 7-hydroxycymopochromanone (PBQ1)*

^1^ H NMR(CDCl_3_, ppm): 1.05, 3 H, s (H-8), 1.20, 3 H, s (H-9), 1.30, 2 H, m (H-6), 1.35, 3 H, s (H-10), 1.61, 2 H, m (H-4), 1.61, 2 H, m (H-5), 2.99, 1 H, s (H-2), 3.00, 1 H, s (H-2), 5.35, 1 H, br s (OH-6^′^), 7.13, 1 H, s (H-4^′^), 7.66, 1 H, s (H-1^′^), 12.00, 1 H, s (OH-7).

^13^ C NMR (CDCl_3_, ppm): 16.3 (C-5), 27.1 (C-10), 28.2 (C-8), 32.2 (C-9), 34.5 (C-4), 36.3 (C-6), 52.7 (C-2), 72.2 (C-7), 73.9 (C-3), 118.3 (C-1^′^), 118.8 (C-5^′^), 120.8 (C-4^′^), 121.1 (C-2^′^), 144.2 (C-6^′^), 156.1 (C-3^′^), 205.4 (C-1).

##### *NMR data for 7-hydroxycymopolone (PBQ2)*

^1^ H NMR (CDCl_3_, ppm):1.28, 6 H, s (H-8, 9), 1.55, 2 H, m (H-6), 1.67, 2 H, m (H-5), 2.20, 3 H, s (H-10), 2.30 2 H, t, *J* = 7.5 Hz (H-4), 6.69, 1 H, s (H-2), 7.18, 1 H, s (H-4^′^), 7.43, s (H-1^′^), 12.32, 1 H, s (OH-7).

^13^ C NMR (CDCl_3_, ppm): 20.5 (C-10), 22.6 (C-5), 29.7 (C-8), 29.7 (C-9), 42.3 (C-4), 43.5 (C-6), 71.5 (C-7), 115.6 (C-1^′^), 119.0 (C-5^′^), 119.8 (C-2), 120.9 (C-2^′^), 121.9 (C-4^′^), 145.0 (C-6^′^), 157.2 (C-3^′^), 162.6 (C-3), 195.9 (C-1).

## Conclusions

The polyisoprenylated bromohydroquinone, PBQ2, was found to affect cell viability of colon cells (HT29) comparable to the chemotherapeutic drug fluorouracil, with selectivity, making this compound an ideal lead candidate suitable for further experimentation in chemotherapy of colon cancer cells. In addition, it showed potent inhibition against CYP1B1 enzyme activities, a marker for cancer and target for drug discovery. Compounds such as PBQ2 that can target both drug metabolizing enzymes and disease state cells are of high value. Such chemotherapeutic and chemopreventive potential implied by the displayed bioactivity validate the on-going search for treatment leads among natural products from endemic tropical biodiversity including marine habitats.

## Competing interests

The authors declare that they have no competing interests.

## Authors’ contribution

SB carried out all CYP inhibition and cell culture assays, WG carried out extraction and purification of natural products, GH designed and assisted all cytotoxicity assays, TJT participated in coordination of cytotoxicity assays, RD conceived of the study and participated in its design and coordination. All authors read and approved final manuscript.
